# CD6 and Syntaxin Binding Protein 6 Variants and Response to Tumor Necrosis Factor Alpha Inhibitors in Danish Patients with Rheumatoid Arthritis

**DOI:** 10.1371/journal.pone.0038539

**Published:** 2012-06-07

**Authors:** Sophine B. Krintel, Laurent Essioux, Assaf Wool, Julia S. Johansen, Ehud Schreiber, Tomer Zekharya, Pinchas Akiva, Mikkel Østergaard, Merete L. Hetland

**Affiliations:** 1 DANBIO Registry and Department of Rheumatology, Copenhagen University Hospital at Glostrup, Copenhagen, Denmark; 2 Hoffmann-La Roche AG, Basel, Switzerland; 3 Compugen Ltd., Tel Aviv, Israel; 4 Department of Medicine, Copenhagen University Hospital at Herlev, University of Copenhagen, Copenhagen, Denmark; 5 Department of Oncology, Copenhagen University Hospital at Herlev, University of Copenhagen, Copenhagen, Denmark; 6 Faculty of Health Sciences, University of Copenhagen, Copenhagen, Denmark; Pasteur Institute of Lille, France

## Abstract

**Background:**

TNFα inhibitor therapy has greatly improved the treatment of patients with rheumatoid arthritis, however at least 30% do not respond. We aimed to investigate insertions and deletions (INDELS) associated with response to TNFα inhibitors in patients with rheumatoid arthritis (RA).

**Methodology and Principal Findings:**

In the DANBIO Registry we identified 237 TNFα inhibitor naïve patients with RA (81% women; median age 56 years; disease duration 6 years) who initiated treatment with infliximab (n = 160), adalimumab (n = 56) or etanercept (n = 21) between 1999 and 2008 according to national treatment guidelines. Clinical response was assessed at week 26 using EULAR response criteria. Based on literature, we selected 213 INDELS potentially related to RA and treatment response using the GeneVa® (Compugen) *in silico* database of 350,000 genetic variations in the human genome. Genomic segments were amplified by polymerase chain reaction (PCR), and genotyped by Sanger sequencing or fragment analysis. We tested the association between genotypes and EULAR good response versus no response, and EULAR good response versus moderate/no response using Fisher’s exact test. At baseline the median DAS28 was 5.1. At week 26, 68 (29%) patients were EULAR good responders, while 81 (34%) and 88 (37%) patients were moderate and non-responders, respectively. A 19 base pair insertion within the CD6 gene was associated with EULAR good response vs. no response (OR = 4.43, 95% CI: 1.99–10.09, p = 7.211×10^−5^) and with EULAR good response vs. moderate/no response (OR = 4.54, 95% CI: 2.29–8.99, p = 3.336×10^−6^). A microsatellite within the syntaxin binding protein 6 (STXBP6) was associated with EULAR good response vs. no response (OR = 4.01, 95% CI: 1.92–8.49, p = 5.067×10^−5^).

**Conclusion:**

Genetic variations within CD6 and STXBP6 may influence response to TNFα inhibitors in patients with RA.

## Introduction

Tumor necrosis factor alpha (TNFα) inhibitor therapy has greatly improved outcome in patients with moderate and severe rheumatoid arthritis (RA) [1]–[3]. Unfortunately, drug response is variable and approximately 30% of patients with RA do not respond to TNFα inhibitors or fail to maintain initial response [1]–[4]. Early and aggressive treatment is essential to suppress inflammation and progression of joint destructions, and as the number of biologic therapies increases, there is a growing need for predictive biomarkers of drug response in patients with RA [5], [6]. Clinical and serological predictors of treatment response such as concomitant methotrexate therapy, functional disability, gender, smoking status, IgM-rheumatoid factor (RF) and antibodies against citrullinated peptides (ACPA) are likely to influence response to TNFα inhibitors. However neither clinical nor serological biomarkers have shown to be useful as predictive biomarkers of response to TNFα inhibitors at the individual level in clinical practice [4], [7], [8].

Since the completion of the Human HapMap Project, there has been a burst in the number of pharmacogenetic studies aiming to identify genetic variation associated with response to TNFα inhibitors in patients with RA. The main focus has been on RA susceptibility genes such as variants within the HLA region, the TNF gene itself and genes implicated in the TNFα signaling pathway [8]–[21]. Recently, two genome-wide association studies (GWASs) investigated the association of hundreds of single nucleotide polymorphisms (SNPs) and response to TNFα inhibitors [22], [23]. So far, several genetic biomarkers with suggestive association have been identified, but results are contradictory or need to be validated.

During the last decade, millions of insertions and deletions (INDELS) have been discovered in human populations and personal genomes. INDELS are structural variations of DNA, owing to deletions or insertions ranging from 1 to 10,000 base pair (bp) in length. Many of these INDELS map to functionally important sites within human genes, and are likely to contribute to phenotypic diversity and diseases including inflammatory diseases such as RA [24], [25]. It is unknown whether INDELS influence treatment response to TNFα inhibitors in patients with RA. The aim of the present study was to investigate the association between INDELS and response to TNFα inhibitors in patients with RA treated in routine care.

## Materials and Methods

### Patients

Based on the DANBIO registry, we identified 237 patients with RA (the Copenhagen Cohort) initiating therapy with TNFα inhibitors between October 1999 and August 2008 at five departments of Rheumatology in the area of Copenhagen, Denmark (Hvidovre Hospital (n = 212), Herlev Hospital (n = 13), Rigshospitalet (n = 7), Bispebjerg Hospital (n = 1), and Gentofte Hospital (n = 4)). The DANBIO registry is a nationwide registry that prospectively collects clinical data on all Danish patients with inflammatory rheumatic joint diseases [26]. Patients were included in the study if they had RA according to the ACR 1987 criteria [27] and had available DNA samples drawn before start of TNFα inhibitor treatment. All patients were naïve to biologic treatment and initiated treatment with TNFα inhibitors (infliximab (n = 160), adalimumab (n = 56), and etanercept (n = 21)) according to the Danish national guidelines. They had high disease activity and/or progressive radiographical structural joint damage despite treatment with at least two different disease-modifying anti-rheumatic drugs (DMARDs) including methotrexate (MTX). Clinical assessments at the start of treatment (baseline) and at week 26 were included in the present study. Clinical evaluation included tender and swollen joint counts (28 joints), visual analogue scales (VAS) scores of pain, patient global and physician global, health assessment questionnaire (HAQ), serum C-reactive protein (CRP), and 28-joint count Disease Activity Score (DAS28) based on 4 variables (the number of swollen and tender joints, patient global score and CRP) [28]. Treatment response was calculated at week 26 using EULAR (European League Against Rheumatism) response criteria [29]. Blood samples for DNA extraction were drawn at baseline and stored at minus 80 degree Celsius.

### Ethics Statement

The study was performed according to the Declaration of Helsinki. Forty-seven patients were also participants in an imaging study [30]. The study was approved by the the Ethical Committee of the Capital Region (Copenhagen), Denmark (H-KH-298094). All patients gave written informed consent to store blood samples in a research biobank for future studies. Written informed consent regarding the use of blood for genetic analysis was waived by the Ethical Committee of the Capital Region (Copenhagen). According to the Danish Health Act, the committee may grant exemption from the requirement of consent if a notifiable database research project does not involve any health risks and if under the given conditions the research project cannot otherwise put a strain on the trial subject. This also applies if it would be impossible or disproportionately difficult to obtain informed consent.

### Marker Selection and Genotyping

We selected genes related to RA and TNFα inhibitor treatment. This included the receptors for TNF and their downstream pathways, genes associated with treatment response in previously published pharmacogenomic studies of patients with RA treated with TNFα inhibitors, genes that were over-expressed in previously published expression studies of patients with RA treated with TNFα inhibitors, RA susceptibility genes, and serological biomarkers for RA. Using the GeneVa® platform (Compugen, Tel-Aviv, Israel), 213 INDELS were chosen according to their confidence level of prediction, the potential effect of the INDEL on the gene and the relevance of the gene. The GeneVa platform incorporates an *in silico* database of approximately 350,000 predicted non-SNP genetic variations, up to a length of 500 bp, in the human genome. DNA was amplified using polymerase chain reaction (PCR). One hundred and twenty-two amplicons were genotyped using sequencing and 91 were genotyped using fragment analysis. When using sequencing, the two genomic copies of the amplicon were sequenced together and separated computationally. SNPs and 1–2 bp INDELS were ignored. Some alleles were grouped together since they could not be reliably separated, for example if the amplicon was long and the sequencing quality became too low. Fragment analysis was used in cases where sequencing could not be applied, usually in the presence of long 1- or 2 bp repeats. The length measurements were up to 1–2 bp, and alleles were grouped together so that there was a minimum difference of 4 bp between groups.

### Statistics

In order to maximize the probability of discovering a response marker we chose to compare the genotypes of EULAR good responders and non-responders, excluding the moderate response group in the initial analysis. In a secondary analysis, the patients with moderate response were added to either the group of good responders or non-responders in order to increase the size of the cohort. The alleles of each amplicon were divided into two groups, and either the dominant or the recessive model for these groups was used. There were two types of allele grouping: all alleles with length smaller or larger than some threshold, or one allele vs. all others. For bi-allelic amplicons there is only one allele grouping possible, one allele vs. the other. There are two tests possible in this case since the recessive and dominant models for one allele are the same as the dominant and recessive models for the other allele, respectively. For multi-allelic amplicons more tests are possible. Only tests for which the minimal genotype group size was at least 10% of the total number of samples with genotypes for this amplicon were considered. The associations between genotypes and EULAR good response versus no response, EULAR good/moderate versus no response, and EULAR good versus moderate/no response were calculated using Fisher’s exact test.

Bonferroni corrections were performed to account for multiple testing. If N_marker_ is the number of amplicons with at least one test possible, and N_test_ is the number of tests for a specific amplicon, then the type I error threshold for any test of a certain amplicon was set at 0.05/(N_marker_ × N_test_). Statistical analysis was performed using R, version 2.6.0 (http://www.R-project.org).

## Results

Baseline characteristics of the 237 patients are shown in Table 1. Median age at inclusion was 56 years, 81% were females, 66% were IgM-RF positive and 57% were anti-cyclic citrullinated protein antibody (anti-CCP) positive. The median DAS28 at baseline was 5.1. A total of 68% initiated treatment with infliximab, 23% with adalimumab, and 9% with etanercept. Eighty-seven % received concomitant MTX treatment. After 26 weeks of treatment, 29% of the patients were classified as good responders, 34% as moderate responders and 37% as non responders according to the EULAR response criteria.

**Table 1:** Demographic and clinical characteristics at baseline.

Variable	All(n = 237)	Good responders(n = 68)	Moderate responders(n = 81)	Non-responders(n = 88)
*Demographics*				
Age, years	56 (19–86)	56 (19–85)	56 (22–86)	56 (19–83)
Women	191 (81%)	56 (82%)	66 (81%)	69 (78%)
Disease duration	6 (0–56)	9 (0–47)	4 (0–47)	6 (0–56)
Ever smokers#	145 (61%)	39 (57%)	54 (68%)	52 (60%)
*Laboratory values*				
IgM-RF positive	157 (66%)	46 (68%)	59 (73%)	52 (59%)
Anti-CCP positive##	70 (57%)	16 (50%)	33 (65%)	21 (54%)
CRP, mg/L	12 (2–280)	16 (4–176)	12 (4–280)	9 (2–134)
*Disease activity* *measures*				
HAQ score (0–3)	1.250 (0–3)	1.125 (0–2.750)	1.250 (0–3)	1.250 (0–2.750)
Pain score (0–100)	57 (2–100)	56.5 (6–97)	62 (8–100)	53 (2–100)
Patient Global score(0–100)	60 (0–100)	52 (13–100)	64 (5–100)	54 (0–100)
Physician’s globalscore (0–100)	48 (0–100)	43.5 (5–100)	51.5 (3–94)	44 (0–95)
DAS28	5.1 (1.6–8.2)	4.9 (3.1–7.4)	5.6 (2.4–8.2)	4.6 (1.6–7.6)
*Treatment*				
Anti TNF drug				
Infliximab	160 (68%)	43 (63%)	52 (64%)	65 (74%)
Etanercept	21 (9%)	5 (7%)	11 (14%)	5 (6%)
Adalimumab	56 (23%)	20 (30%)	18 (22%)	18 (20%)
Glucocorticoids	66 (28%)	19 (28%)	24 (30%)	23 (26%)
Methotrexate	193 (81%)	56 (82%)	67 (83%)	70 (80%)
Methotrexate dose,mg/week	20 (0–25)	22.5 (0–25)	20 (0–25)	20 (0–25)

Values are given as median (range) or number (percentage of total).

# 3 patients had missing smoking status.

## 115 patients had missing anti-CCP values.

A total of 213 amplicons were tested. Detailed information regarding the tested amplicons including number of alleles for each amplicon, number of tests when comparing good responders and non-responders, length difference between longest and shortest allele, rate (%) of the samples that were successfully genotyped, Hardy-Weinberg equilibrium, and p-values (Fisher’s exact test) comparing good responders to non-responders is listed in Table S1. Two amplicons failed genotyping and another amplicon turned out to be non-variable. In 19 of the tested amplicons, at least 5% of the samples had no genotype (rate <95%), and in 5 of these amplicons at least 10% of the samples had no genotype (rate <90%). The lowest genotyping rate for an amplicon was 72%. Missing genotypes could be the result of an unanticipated SNP in one of the primers. No correlation between genotyping rates and genomic location was observed. Seventy-one bi-allelic amplicons and 139 multi-allelic amplicons were identified. There were 202 amplicons for which N_test_ was at least one; hence the number of markers (N_marker_) was 202. Two amplicons had a p-value less than the significance threshold. One was the bi-allelic CGEN-40002 and the other was the multi-allelic CGEN-40003.

### Patients with Good or no EULAR Response

#### CGEN-40002

The bi-allelic CGEN-40002 amplicon represents a 19 bp insertion within the CD6 gene on chromosome 11. The lengths of the alleles are 493 and 512 bp respectively. Forty-five patients out of 156 (29%) with either EULAR good response or EULAR no response were positive for the 512 allele. Imposing a 10% group size condition in which each genotype group consisted of a minimum of 10% of all the patients, only one test was possible (the existence of the long 512 allele), and the significance threshold was then 0.05/(202×1)  = 2.475×10^−4^. Patients with the 512 allele were more likely to achieve a EULAR good response than no response (Odds ratio (OR)  = 4.43, p = 7.211×10^−5^), Table 2. The power was 59% using the significance threshold of 2.475×10^−4^ and 10,000 simulations.

**Table 2:** Association between genotype and EULAR good response versus EULAR no response.

	Good EULARresponse (n = 68)	No EULARresponse (n = 68)	Significancethreshold***	p-value	OddsRatio
**CGEN-40002**					
Allele 512					
negative	37	74	2.475×10^−4^	7.211×10^−5^	4.43*
Allele 512					
positive	31	14			
**CGEN-40003**					
Both alleles ≤280	50	36	9.067×10^−5^	5.067×10^−5#^	4.01**
One allele>280	18	52			

* Odds ratio for EULAR good response being 512 positive; #adjusted p-value;

** Odds ratio for EULAR good response when both alleles are ≤280;

*** after correction for dependency.

#### CGEN-40003

CGEN-40003 represents a multi-allelic amplicon located within the syntaxin binding protein 6 (STXBP6) gene on chromosome 14. It has seven alleles ranging in lengths between 252 and 294 bp. The distribution of genotype within the 156 patients with either EULAR good response or EULAR no response is shown in Table 3. Imposing the 10% group size condition, 8 tests were possible (N_test_ = 8), Table 4. Assuming independence of the 8 tests, the significance threshold was 0.05/(202×8)  = 3.094×10^−5^. Test #2 achieved the best result, shown in Table 2. Patients with two alleles ≤280 bp were more likely to achieve a EULAR good response than no response (OR  = 4.01, p = 5.067×10^−5^) compared to patients with one or two alleles >280 bp. The p-value for this test was higher than the significance threshold. However, the eight tests are not independent. Inspection showed that tests #4 and #6 are identical and tests #1 and #5 are almost identical. Therefore, we performed a simulation to assess the actual probability to get such a p-value as a best result of these 8 tests. In each round the response parameter was randomly permuted between the samples and the tests were performed recording the minimal p-value attained. After 600,000 rounds there were 83 results lower than or equal to 5.067×10^−5^, corresponding to an effective N_test_ of 2.73 ( = [83/600000]/5.067×10^−5^). The adjusted significance threshold was then 0.05/(202×2.73)  = 9.067×10^−5^, larger than the p-value. The power was 55% using the adjusted significance threshold and 10,000 simulations.

**Table 3:** Genotype distribution of the CGEN-40003 amplicon in patients with EULAR good or no response.

Genotype	Patients with EULAR goodor no response(n = 156)*
252/252	0
252/271	1
252/275	0
252/280	0
252/284	0
252/288	0
252/294	0
271/271	79
271/275	0
271/280	4
271/284	19
271/288	33
271/294	3
275/275	0
275/280	1
275/284	1
275/288	2
275/294	1
280/280	1
280/284	0
280/288	4
280/294	0
284/284	1
284/288	3
284/294	0
288/288	3
288/294	0
294/294	0

* Values are numbers.

**Table 4:** Possible tests imposing a 10% genotype group size condition in the 156 patients with either EULAR good response or no response.

Possible tests	Positive n (%)	Negative n (%)
#1 Both alleles≤271	80 (51%)	76 (49%)
# 2 Both alleles≤280	86 (55%)	70 (45%)
# 3 Both alleles≤284	107 (69%)	49 (31%)
# 4 One allele≤271	139 (89%)	17 (11%)
# 5 Both alleles = 271	79 (51%)	77 (49%)
# 6 One allele = 271	139 (89%)	17 (11%)
# 7 One allele = 284	24 (15%)	132 (85%)
# 8 One allele = 288	45 (29%)	111 (71%)

### Patients with Good, Moderate or no EULAR Response

In the secondary analysis, patients with EULAR moderate response at week 26 were added to the analysis to increase the number of patients and hence the power to identify INDELS associated with response to TNFα inhibitors. When adding the moderate responders to the non-responders, only CGEN-40002 was associated with response. No amplicons, including CGEN-40003 and CGEN-40002, were associated with response when good responders were combined with moderate responders.

For patients with moderate response, the distribution of the CGEN-40002 alleles was very similar to patients with no response. Sixty-seven patients did not have the 512 allele and 12 patients were either heterozygote or homozygote for the 512 allele (two patients had no genotype available). Patients positive for the 512 allele were more likely to achieve a EULAR good response than moderate/no response compared to patients negative for the 512 allele (OR = 4.54, p = 3.336×10^−6^), Table 5. If N_test_ = 3 (no moderates added, moderates added to good response, moderates added to no response), the significance threshold was 8.251×10^−5^, and the power was 74.5% after 10,000 simulations.

**Table 5:** Association between genotype and EULAR good response versus EULAR moderate response/no response.

	Good EULARresponse (n = 68)	Moderate/no response(n = 167)	Significancethreshold***	p-value	OddsRatio
**CGEN-40002**					
Allele 512					
negative	37	141	8.251×10^−5^	3.336×10^−6^	4.54*
Allele 512					
positive	31	26			

* Odds ratio for EULAR good response being 512 positive,

*** after correction for dependency.

**Figure 1:**
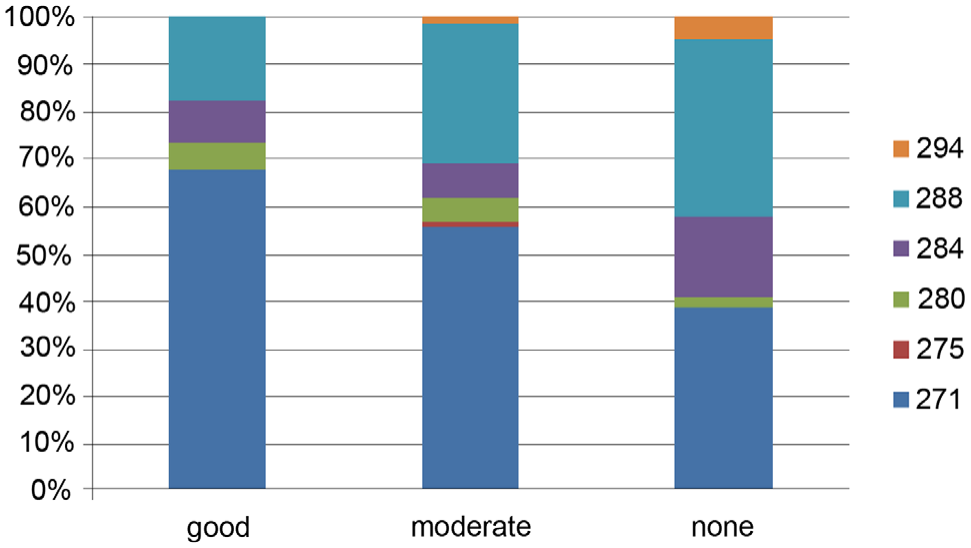
Allele distribution of the CGEN-40003 amplicon according to EULAR response. The Y-axis indicates percentage of patients. The X-axis indicates EULAR response (good, moderate, none). The colored boxes indicate the size (base pair) of the longest allele.

Figure 1 shows the distribution of the CGEN-40003 alleles according to EULAR response. Patients with the longest CGEN-40003 alleles were more often classified as non- and moderate responders. To investigate the relationship between allele distribution and EULAR response, we compared the maximal allele length with change in DAS28 scores after 26 weeks of treatment (delta DAS). The Spearman rank correlation and the Pearson correlation were approximately −0.25 with a p-value of 2.98×10^−5^ and 5.29×10^−5^, respectively.

## Discussion

Our main finding is that INDELS within the CD6 gene and the STXBP6 gene are associated with EULAR response to TNFα inhibitor treatment in patients with RA treated in routine care. During the last decade biologic therapies have greatly improved the treatment of patients with RA. Although highly effective, concerns remain about the variable response rates to TNFα inhibitors, the high costs of these drugs, and the risk of adverse events associated with their use. Identification of genetic markers associated with response to biologic therapies would allow tailored treatments to individual patients with RA.

In this study of patients with RA we identified two genetic biomarkers that are statistically associated with response to TNFα inhibitor therapy. The CGEN-40002 amplicon is significantly associated with EULAR good response versus EULAR no response and EULAR good response versus EULAR moderate/no response. The CGEN-40002 amplicon represents an insertion within the CD6 gene on chromosome 11. The variation can be found in version 130 of dbSNP, entry rs55799216. The genomic location of the insertion is at position 60,542,552 on chromosome 11 (NCBI build 36), the sequence alignment of the two alleles is:

TCGCTCAAGGGGAAAAGGAGAAAGGAAGGG- - - - - - - - - - - - - - - - - - -TAAAAGAAGA

TCGCTCAAGGGGAAAAGGAGAAAGGAAGGGGAAAAGGAGAAAGGAAGGGTAAAAGAAGA

The inserted segment in the long allele is a copy of the preceding 19 bp of the short allele. The variation is located in the last intron of the gene, about 100 bp downstream of the end of the preceding exon. This exon is alternative according to RNA evidence, and there is also RNA evidence of intron retention for this intron, both in human and in chimpanzee. Noteworthy, both the human expressed sequence tags (ESTs) that cross this exon-intron junction contain the 19 bp insertion. The 19 bp variation may alter the splicing of the CD6 gene. CD6 is a cell surface receptor which belongs to the scavenger receptor cysteine-rich (SRCR) protein superfamily (SRCRSF) and is expressed primarily on thymocytes and lymphocytes [31], [32]. CD6 is considered to play an important role in lymphocyte development and activation through its binding to the activated leukocyte cell adhesion molecule ALCAM/CD166. Interaction between CD6 and ALCAM is crucial for proper immunological synapse maturation and T cell proliferative responses [32]. A recent study suggested that the CD6-ALCAM interaction activates the three mitogen-activated protein kinase (MAPK) cascades which are suggested to influence apoptosis of lymphocytes [33]. Interesting, genetic variants within the MAPK genes are also suggested to influence response to TNFα inhibitors in patients with RA [34], [35].

The CGEN-40003 amplicon is significantly associated with EULAR good response compared to EULAR no response. This variation is an insertion with 7 alleles of varying lengths, and we found that patients with the longest CGEN-40003 alleles were more often classified as non- and moderate responders. The CGEN-40003 amplicon represents a 2 bp short repeat within the STXBP6 gene (syntaxin binding protein 6) on chromosome 14. The variation lies within a very long intron, approximately 10 Kbp away from the closest exon. This microsatellite is represented in dbSNP version 130 by several entries, for example rs10630160, rs34132743, rs35668825. The reference genome contains 17 TG repeats in positions 24,503,694-24,503,728, and dbSNP describes alleles with 1–9 additional TG repeats. Adjacent to the variation is a possible transcription binding site for Peroxisome proliferator-activated receptor gamma (PPARG), and a highly conserved 250 bp long genomic region with a high Evolutionary and Sequence Pattern Extraction through Reduced Representations (ESPERR) score suggesting a potential functional element in the genome [36]. The variation may have a direct effect on the transcription of the STXBP6 gene or be correlated with another variation in the region. STXBP6 is not a known RA susceptibility gene or biomarker of response to TNFα inhibitors in patients with RA but in a previous microarray study of patients with RA treated with TNFα inhibitors, STXBP6 was differentially expressed in responders compared to non responders [37]. STXBP6 is a syntaxin binding protein that binds to components of the SNARE complex which inhibits membrane fusions including phagocytosis [38]. Interestingly, a combined GWAS and replication study has recently identified 4 loci within STXBP6, ITGA4, MLZE and the MHC region associated with monocyte counts in a cohort of 14,792 Japanese individuals [39]. STXBP6 is suggested to play a role in the immune response by altering the ability of phagocytosis and antigen presentation of monocytes and macrophages [39].

In our primary analysis, we tested the allele frequency in patients with good EULAR response versus no EULAR response. Our basic assumption was that for a biomarker which is correlated to response there should be two distinct distributions of response for the two genotypic groups. The moderate responders are likely to be in the middle with contributions from both genotypic groups. Therefore adding the moderate responders to either the good or the non-responders was likely to reduce the p-value. On the other hand adding the 81 patients with moderate EULAR response increased the size of the population tested, making it possible for less extreme distributions to reach significance. When patients with moderate response were added, only CGEN-40002 was associated with EULAR good response versus moderate/no EULAR response. The CGEN-40003 amplicon was not associated with EULAR response when patients with moderate EULAR response were added to patients with either good or no response. However, the genotypic distributions of patients with moderate response were in between two extreme groups suggesting a quantitative response effect of this gene.

Some strengths of our study are the genetically homogenous and well-characterized cohort of Danish patients with RA treated with TNFα inhibitors in routine care. Clinical data were collected prospectively and independently of the present study in the DANBIO registry, which has high data completeness and coverage (>90%) [26]. One may argue that the population heterogeneity with regard to disease duration, HAQ, and number of previous DMARDs is a limitation of the study since long disease duration and severe joint destruction may influence treatment response and the assessment of clinical response. However, heterogeneity also increases the external validity of the findings.

In recent years, candidate gene studies and GWASs have successfully identified RA susceptibility genes. Similarly, several genetic loci with suggestive association with response to TNFα inhibitors have been identified, but only few have been replicated in independent cohorts. This inconsistency may be influenced by several factors including small sample size, small effect size of the identified loci, allele frequency, heterogeneity with regard to baseline characteristics, and differences in outcome measurements, disease severity and TNFα inhibitor therapy. Lack of statistical power remains one of the great challenges in pharmacogenetic studies [40]–[42]. Small sample size can provide incorrect estimation of effect size and small sample size of an initial study might also explain the lack of replication in independent cohorts [21], [42]–[43]. In our study, the sample size of 237 patients does not allow for the significance to be incontestable. Therefore, the findings need to be validated in other populations of patients with RA treated with TNFα inhibitors. Different classes of TNF inhibitors (TNF-soluble receptors and neutralizing anti-TNF antibodies) may not act through shared biological pathways which warrant studies to be restricting to only one drug class or even to a single drug [42]. Our study was not designed to perform sub-analyses on patients stratified according to TNFα inhibitor drugs, but this would be an interesting approach in a validation study.

The majority of the currently practiced association studies utilize SNP based technologies for the genotyping process. Such methods, which genotype numerous SNPs, usually require a large sample collection in order to achieve statistically significant results. However, it might be difficult to achieve significant results when using a relatively small sample set. Such difficulty can be resolved by genotyping a relatively small number of carefully selected genetic variations rather than using the genome-wide approach [44], [45]. Like SNPs, INDELS can also be used for association studies. Yet, an advantage of using INDELS is that, since they represent greater structural variation than SNPs, they are more likely to be the actual genetic basis of phenotypic differences between individuals. Moreover, in some cases utilizing multi-allelic INDELS (such as microsatellites) might be more useful than SNPs [46]. In the present study, we did only test common INDELS with a length of maximum 500 bp. Rare INDELS >500 kb and other large genetic variations such as copy number variations, which are likely to be more deleterious, were not tested.

In conclusion, we identified genetic variations within the CD6 and the STXBP6 genes associated with response to TNFα inhibitors in a cohort of homogeneous Danish patients with RA. Future, prospective studies including a larger number of patients with RA are needed to achieve a greater understanding of treatment failure and to evaluate if genetic variations within the CD6 and the STXBP6 can provide useful information regarding treatment response and contribute to a more personalized treatment strategy in the future.

## Supporting Information

Table S1 **Tested amplicons.** Protocol: sequencing or fragment analysis; #alleles: the number of alleles for each INDEL; Ntest: number of tests that were performed for each INDEL when comparing good responders and non-responders; Length range: length difference between longest and shortest allele; Rate (%): the fraction of samples that were genotyped successfully; Hw (Hardy-Weinberg equilibrium): p-value of a chi-square test comparing the homozygotes and heterozygotes distribution. The value in the table is the minimal value for all subdivisions of the alleles into two groups; Min p-value: p-value of a Fisher's exact test comparing good responders to non-responders. The value in the table is the minimal value of all tests done for each INDEL.
(DOC)
