# Supplementary material for: CD6 and Syntaxin Binding Protein 6 Variants and Response to Tumor Necrosis Factor Alpha Inhibitors in Danish Patients with Rheumatoid Arthritis
Source: PLoS One. 2012 Jun 7;7(6):e38539. doi: 10.1371/journal.pone.0038539 (PMC3369852; doi:10.1371/journal.pone.0038539)
Supplement: Table S1 — Tested amplicons. Protocol: sequencing or fragment analysis; #alleles: the number of alleles for each INDEL; Ntest: number of tests that were performed for each INDEL when comparing good responders and non-responders; Length range: length difference between longest and shortest allele; Rate (%): the fraction of samples that were genotyped successfully; Hw (Hardy-Weinberg equilibrium): p-value of a chi-square test comparing the homozygotes and heterozygotes distribution. The value in the table is the minimal value for all subdivisions of the alleles into two groups; Min p-value: p-value of a Fisher's exact test comparing good responders to non-responders. The value in the table is the minimal value of all tests done for each INDEL. (DOC) [file pone.0038539.s001.doc]

Table S1. Tested amplicons

| Amplicon | Protocol | Comment | #alleles | length range | Ntest | rate | hw | min p-value |
| --- | --- | --- | --- | --- | --- | --- | --- | --- |
| GVAR1002 | Sequencing |  | 2 | 37 | 2 | 91.98312 | 0.3496615 | 0.7053017 |
| GVAR1003 | Fragment Analysis |  | 8 | 35 | 14 | 100 | 0.000699007 | 0.1839306 |
| GVAR1004 | Sequencing |  | 9 | 32 | 14 | 94.51477 | 0.0307812 | 0.1315147 |
| GVAR1008 | Sequencing |  | 8 | 24 | 13 | 92.40506 | 0.4833589 | 0.0265306 |
| GVAR1009 | Sequencing | failed |  |  | 0 |  |  |  |
| GVAR1011 | Sequencing |  | 2 | 32 | 1 | 99.15612 | 0.812525 | 0.2368393 |
| GVAR1012 | Fragment Analysis |  | 6 | 28 | 11 | 99.57806 | 0.4593001 | 0.2572754 |
| GVAR1013 | Fragment Analysis |  | 10 | 44 | 14 | 99.57806 | 0.002028418 | 0.0779421 |
| GVAR1014 | Fragment Analysis |  | 7 | 29 | 12 | 99.15612 | 0.000216249 | 0.8352255 |
| GVAR1015 | Sequencing |  | 2 | 262 | 2 | 97.8903 | 0.9449062 | 0.0592848 |
| GVAR1016 | Fragment Analysis |  | 6 | 175 | 0 | 96.62447 | 1 |  |
| GVAR1017 | Sequencing |  | 2 | 16 | 1 | 97.8903 | 0.9620637 | 0.8717897 |
| GVAR1018 | Sequencing |  | 2 | 51 | 1 | 99.15612 | 0.2753894 | 0.1644283 |
| GVAR1019 | Sequencing |  | 2 | 177 | 1 | 100 | 0.9687276 | 0.62326 |
| GVAR1020 | Sequencing |  | 2 | 16 | 1 | 98.73418 | 0.5555237 | 0.5763814 |
| GVAR1021 | Sequencing |  | 15 | 56 | 19 | 90.7173 | 0.04107247 | 0.0640693 |
| GVAR1022 | Fragment Analysis |  | 5 | 40 | 6 | 99.57806 | 0.4888462 | 0.6201928 |
| GVAR1024 | Fragment Analysis |  | 9 | 32 | 17 | 97.46835 | 7.88E-05 | 0.0255922 |
| GVAR1025 | Sequencing |  | 2 | 4 | 2 | 98.31224 | 0.9982633 | 0.8313935 |
| GVAR1028 | Fragment Analysis |  | 4 | 21 | 4 | 100 | 0.9076551 | 0.6311735 |
| GVAR1031 | Fragment Analysis |  | 9 | 64 | 16 | 99.15612 | 0.458447 | 0.0147142 |
| GVAR1032 | Sequencing |  | 2 | 3 | 2 | 97.46835 | 0.8029823 | 0.8452794 |
| GVAR1035 | Fragment Analysis |  | 8 | 45 | 13 | 94.9367 | 2.09E-05 | 0.016184 |
| GVAR1036 | Sequencing |  | 5 | 160 | 11 | 95.7806 | 0.3191389 | 0.0479387 |
| GVAR1037 | Sequencing |  | 2 | 12 | 1 | 98.31224 | 0.9488566 | 0.3785425 |
| GVAR1038 | Sequencing |  | 3 | 24 | 2 | 97.8903 | 0.3470397 | 1 |
| GVAR1039 | Fragment Analysis |  | 3 | 10 | 2 | 99.15612 | 0.9875348 | 0.1613171 |
| GVAR1042 | Sequencing |  | 2 | 25 | 1 | 99.57806 | 0.5940512 | 0.8629651 |
| GVAR1043 | Fragment Analysis |  | 7 | 26 | 10 | 98.31224 | 0.3948816 | 0.0730158 |
| GVAR1044 | Fragment Analysis |  | 8 | 27 | 13 | 99.57806 | 8.32E-07 | 0.0091174 |
| GVAR1045 | Sequencing |  | 8 | 28 | 14 | 99.15612 | 0.3685957 | 0.3026672 |
| GVAR1046 | Sequencing |  | 2 | 18 | 1 | 99.57806 | 0.5321876 | 1 |
| GVAR1050 | Sequencing |  | 9 | 44 | 16 | 91.13924 | 0.3149599 | 0.0354219 |
| GVAR1051 | Sequencing |  | 5 | 16 | 10 | 95.7806 | 0.06885812 | 0.1499738 |
| GVAR1054 | Fragment Analysis |  | 8 | 39 | 13 | 100 | 0.02022856 | 0.0731716 |
| GVAR1055 | Sequencing |  | 2 | 18 | 2 | 97.8903 | 0.4114265 | 0.2547327 |
| GVAR1058 | Fragment Analysis |  | 5 | 22 | 7 | 98.73418 | 0.07726842 | 0.120449 |
| GVAR1060 | Fragment Analysis |  | 6 | 22 | 10 | 100 | 0.2461011 | 0.1839306 |
| GVAR1061 | Fragment Analysis |  | 7 | 27 | 7 | 98.73418 | 0.000200558 | 0.5837407 |
| GVAR1062 | Sequencing |  | 2 | 19 | 2 | 75.94937 | 0.623919 | 0.3480949 |
| GVAR1066 | Sequencing |  | 2 | 6 | 2 | 97.8903 | 0.9292298 | 1 |
| GVAR1068 | Sequencing |  | 2 | 73 | 2 | 98.73418 | 0.06645694 | 0.0461888 |
| GVAR1069 | Fragment Analysis |  | 2 | 318 | 1 | 97.8903 | 0.000180369 | 0.6856705 |
| GVAR1070 | Fragment Analysis |  | 12 | 129 | 19 | 98.73418 | 0.005172171 | 0.1977418 |
| GVAR1072 | Sequencing |  | 4 | 177 | 6 | 93.67089 | 0.9874521 | 0.0042536 |
| GVAR1074 | Sequencing |  | 2 | 5 | 1 | 98.73418 | 0.9931665 | 0.8659354 |
| GVAR1075 | Sequencing |  | 2 | 16 | 0 | 99.15612 | 1 |  |
| GVAR1077 | Fragment Analysis |  | 4 | 14 | 4 | 99.57806 | 0.2758729 | 0.0635679 |
| GVAR1079 | Sequencing |  | 2 | 4 | 2 | 95.35865 | 5.50E-06 | 0.0163921 |
| GVAR1080 | Sequencing |  | 3 | 4 | 2 | 97.04641 | 0.4080341 | 0.5989624 |
| GVAR1081 | Sequencing |  | 2 | 11 | 1 | 96.62447 | 0.9764386 | 0.4150061 |
| GVAR1082 | Sequencing |  | 6 | 83 | 3 | 99.57806 | 0.1746744 | 1 |
| GVAR1084 | Sequencing |  | 3 | 88 | 1 | 98.31224 | 0.6578258 | 1 |
| GVAR1085 | Sequencing |  | 2 | 2 | 1 | 96.62447 | 0.8542351 | 0.5135962 |
| GVAR1086 | Sequencing |  | 2 | 171 | 1 | 100 | 0.5974929 | 0.744847 |
| GVAR1087 | Sequencing |  | 2 | 30 | 1 | 98.73418 | 0.9425622 | 0.3291439 |
| GVAR1090 | Fragment Analysis |  | 6 | 26 | 8 | 99.57806 | 8.61E-07 | 0.1035664 |
| GVAR1092 | Sequencing |  | 3 | 8 | 2 | 97.04641 | 0.9442787 | 0.7462101 |
| GVAR1094 | Fragment Analysis |  | 5 | 29 | 3 | 98.73418 | 0.4876946 | 0.4209604 |
| GVAR1095 | Fragment Analysis |  | 8 | 49 | 12 | 100 | 0.02173728 | 0.5763138 |
| GVAR1097 | Sequencing |  | 14 | 64 | 21 | 86.49789 | 0.1683338 | 0.1203212 |
| GVAR1101 | Fragment Analysis |  | 2 | 4 | 1 | 99.15612 | 0.1455989 | 0.0176099 |
| GVAR1102 | Fragment Analysis |  | 4 | 23 | 8 | 90.7173 | 0.2419322 | 0.0946656 |
| GVAR1103 | Sequencing |  | 3 | 230 | 3 | 90.7173 | 3.64E-19 | 0.4276849 |
| GVAR1104 | Sequencing |  | 2 | 4 | 1 | 98.31224 | 0.2508073 | 0.870195 |
| GVAR1105 | Sequencing |  | 2 | 0 | 1 | 97.8903 | 0.9571 | 1 |
| GVAR1106 | Fragment Analysis |  | 4 | 24 | 1 | 100 | 0.6298806 | 0.6045449 |
| GVAR1108 | Sequencing |  | 2 | 6 | 2 | 88.6076 | 0.7095354 | 0.3447421 |
| GVAR1109 | Sequencing |  | 2 | 4 | 1 | 98.31224 | 0.9756756 | 0.20724 |
| GVAR1110 | Sequencing |  | 2 | 35 | 2 | 96.20253 | 0.2623917 | 0.4137425 |
| GVAR1111 | Fragment Analysis |  | 7 | 24 | 7 | 72.1519 | 1.74E-24 | 0.5979502 |
| GVAR1112 | Sequencing |  | 2 | 70 | 1 | 100 | 0.5725584 | 1 |
| GVAR1113 | Sequencing |  | 3 | 16 | 5 | 98.73418 | 0.8305394 | 0.5073025 |
| GVAR1114 | Fragment Analysis |  | 6 | 26 | 10 | 100 | 8.99E-06 | 0.3284014 |
| GVAR1115 | Sequencing |  | 2 | 45 | 2 | 97.04641 | 0.217646 | 0.443757 |
| GVAR1116 | Fragment Analysis |  | 5 | 97 | 3 | 98.73418 | 0.6226307 | 0.2190297 |
| GVAR1118 | Sequencing |  | 27 | 87 | 24 | 99.15612 | 0.2221165 | 0.408046 |
| GVAR1119 | Fragment Analysis | failed |  |  | 0 |  |  |  |
| GVAR1122 | Sequencing |  | 2 | 24 | 1 | 99.15612 | 0.9826915 | 0.4159516 |
| GVAR1124 | Fragment Analysis |  | 5 | 17 | 7 | 99.57806 | 0.4101683 | 0.5165825 |
| GVAR1125 | Fragment Analysis |  | 8 | 29 | 13 | 97.46835 | 0.000760787 | 0.4104837 |
| GVAR1128 | Fragment Analysis |  | 15 | 83 | 25 | 99.15612 | 0.00623105 | 0.0372309 |
| GVAR1130 | Fragment Analysis |  | 2 | 10 | 2 | 97.46835 | 0.4946642 | 0.0463434 |
| GVAR1131 | Fragment Analysis |  | 5 | 19 | 3 | 100 | 0.08212266 | 0.3332501 |
| GVAR1134 | Sequencing |  | 2 | 6 | 1 | 96.62447 | 1.45E-10 | 0.4005987 |
| GVAR1135 | Fragment Analysis |  | 14 | 218 | 6 | 99.57806 | 0.02860637 | 0.7161757 |
| GVAR1136 | Sequencing |  | 5 | 24 | 7 | 96.62447 | 7.87E-07 | 0.4278857 |
| GVAR1139 | Fragment Analysis |  | 5 | 17 | 6 | 97.8903 | 0.131053 | 0.2517852 |
| GVAR1143 | Sequencing |  | 3 | 16 | 2 | 99.15612 | 0.5232726 | 0.337605 |
| GVAR1149 | Sequencing | not variable | |  | 0 |  |  |  |
| GVAR1150 | Fragment Analysis |  | 5 | 36 | 6 | 100 | 0.5579647 | 0.5252748 |
| GVAR1151 | Fragment Analysis |  | 12 | 90 | 20 | 96.62447 | 0.3551687 | 0.1426106 |
| GVAR1152 | Fragment Analysis |  | 5 | 19 | 2 | 97.8903 | 0.5744917 | 0.5126939 |
| GVAR1153 | Sequencing |  | 2 | 16 | 0 | 98.73418 | 1 |  |
| GVAR1155 | Fragment Analysis |  | 10 | 43 | 12 | 99.57806 | 0.000173264 | 0.3039997 |
| GVAR1157 | Fragment Analysis |  | 4 | 20 | 5 | 99.15612 | 2.42E-07 | 0.819151 |
| GVAR1159 | Fragment Analysis |  | 6 | 38 | 8 | 99.15612 | 0.5254088 | 0.2085571 |
| GVAR1161 | Sequencing |  | 3 | 51 | 5 | 99.57806 | 0.09815247 | 0.1707072 |
| GVAR1163 | Fragment Analysis |  | 4 | 27 | 3 | 99.15612 | 0.9595633 | 0.2879475 |
| GVAR1164 | Fragment Analysis |  | 4 | 21 | 5 | 99.57806 | 4.81E-06 | 0.5374373 |
| GVAR1165 | Fragment Analysis |  | 12 | 463 | 15 | 94.9367 | 5.96E-29 | 0.4634712 |
| GVAR1166 | Sequencing |  | 2 | 16 | 1 | 100 | 0.9962981 | 0.2429 |
| GVAR1170 | Fragment Analysis |  | 8 | 30 | 10 | 99.57806 | 0.1363586 | 0.3908245 |
| GVAR1172 | Sequencing |  | 2 | 4 | 2 | 97.8903 | 0.3473349 | 0.2029508 |
| GVAR1176 | Fragment Analysis |  | 4 | 19 | 4 | 98.31224 | 0.1618521 | 0.0614244 |
| GVAR1177 | Sequencing |  | 2 | 21 | 2 | 97.04641 | 0.8646481 | 0.2119612 |
| GVAR1178 | Sequencing |  | 2 | 118 | 2 | 98.73418 | 0.08874323 | 0.1442585 |
| GVAR1182 | Sequencing |  | 6 | 18 | 10 | 94.9367 | 0.3263351 | 0.1641529 |
| GVAR1184 | Sequencing |  | 8 | 36 | 14 | 98.31224 | 0.3082874 | 0.1829132 |
| GVAR1185 | Fragment Analysis |  | 14 | 60 | 21 | 98.73418 | 2.78E-06 | 0.1483571 |
| GVAR1186 | Fragment Analysis |  | 9 | 78 | 0 | 99.57806 | 1 |  |
| GVAR1187 | Sequencing |  | 2 | 12 | 2 | 94.09283 | 0.6855246 | 0.2229537 |
| GVAR1188 | Fragment Analysis |  | 2 | 6 | 1 | 99.15612 | 0.263622 | 0.6264871 |
| GVAR1189 | Fragment Analysis |  | 4 | 49 | 5 | 98.31224 | 0.03320572 | 0.0464067 |
| GVAR1192 | Fragment Analysis |  | 4 | 15 | 5 | 97.8903 | 0.002641364 | 0.278613 |
| GVAR1193 | Fragment Analysis |  | 2 | 4 | 1 | 97.46835 | 0.142238 | 0.3990886 |
| GVAR1194 | Sequencing |  | 3 | 80 | 4 | 98.31224 | 0.3900883 | 0.6553724 |
| GVAR1195 | Sequencing |  | 2 | 30 | 1 | 100 | 0.7483032 | 0.8120629 |
| GVAR1196 | Fragment Analysis |  | 5 | 17 | 11 | 98.73418 | 0.003575904 | 0.0727263 |
| GVAR1197 | Fragment Analysis |  | 12 | 44 | 14 | 98.73418 | 0.1934605 | 0.1060696 |
| GVAR1198 | Sequencing |  | 3 | 8 | 3 | 86.07595 | 0.5717968 | 0.5960776 |
| GVAR1199 | Sequencing |  | 2 | 32 | 1 | 99.57806 | 0.5574046 | 0.2629928 |
| GVAR1201 | Fragment Analysis |  | 9 | 44 | 15 | 97.46835 | 0.001737804 | 0.0446981 |
| GVAR1202 | Fragment Analysis |  | 9 | 34 | 15 | 99.57806 | 0.1395513 | 0.0463821 |
| GVAR1204 | Sequencing |  | 2 | 421 | 1 | 99.57806 | 0.5394087 | 0.0962418 |
| GVAR1205 | Sequencing |  | 5 | 16 | 6 | 98.31224 | 0.7098447 | 0.3829376 |
| GVAR1206 | Fragment Analysis |  | 9 | 39 | 12 | 97.46835 | 1.25E-06 | 0.0324133 |
| GVAR1207 | Sequencing |  | 2 | 21 | 1 | 99.57806 | 0.669641 | 1 |
| GVAR1209 | Sequencing |  | 2 | 25 | 2 | 99.57806 | 0.9746814 | 0.0191814 |
| GVAR1211 | Fragment Analysis |  | 4 | 41 | 3 | 99.15612 | 0.004689088 | 1 |
| GVAR1212 | Sequencing |  | 2 | 4 | 1 | 100 | 0.06707983 | 0.2150262 |
| GVAR1214 | Sequencing |  | 5 | 126 | 7 | 98.31224 | 3.07E-13 | 0.5346056 |
| GVAR1220 | Sequencing |  | 2 | 2 | 1 | 99.15612 | 0.7430942 | 0.1786587 |
| GVAR1221 | Sequencing |  | 2 | 16 | 1 | 99.57806 | 0.9050944 | 0.258564 |
| GVAR1222 | Sequencing |  | 2 | 28 | 0 | 99.57806 | 1 |  |
| GVAR1223 | Fragment Analysis |  | 5 | 27 | 7 | 99.57806 | 5.26E-08 | 0.1094363 |
| GVAR1228 | Sequencing |  | 3 | 40 | 2 | 97.8903 | 0.9588724 | 0.0770215 |
| GVAR1229 | Sequencing |  | 6 | 140 | 11 | 98.73418 | 0.005943404 | 0.0042417 |
| GVAR1230 | Sequencing |  | 7 | 198 | 5 | 97.8903 | 0.3023265 | 0.1379129 |
| GVAR1231 | Sequencing |  | 3 | 42 | 2 | 99.15612 | 0.877724 | 0.6294333 |
| GVAR1233 | Fragment Analysis |  | 13 | 101 | 8 | 95.7806 | 8.71E-33 | 0.729164 |
| GVAR1234 | Fragment Analysis |  | 6 | 46 | 8 | 99.57806 | 0.1300582 | 0.2004731 |
| GVAR1236 | Fragment Analysis |  | 6 | 22 | 8 | 100 | 1.15E-18 | 0.697905 |
| GVAR1237 | Sequencing |  | 9 | 32 | 14 | 99.15612 | 0.2530233 | 0.2029508 |
| GVAR1238 | Sequencing |  | 2 | 5 | 1 | 98.73418 | 0.5435271 | 0.7429359 |
| GVAR1239 | Fragment Analysis |  | 9 | 38 | 15 | 100 | 0.1413304 | 0.0003817 |
| GVAR1245 | Fragment Analysis |  | 9 | 39 | 16 | 100 | 2.59E-05 | 0.1354765 |
| GVAR1246 | Sequencing |  | 2 | 60 | 1 | 97.8903 | 0.888414 | 0.0406351 |
| GVAR1248 | Fragment Analysis |  | 7 | 33 | 11 | 99.57806 | 0.000224228 | 0.2246114 |
| GVAR1249 | Fragment Analysis |  | 10 | 50 | 13 | 100 | 0.0255757 | 0.1768747 |
| GVAR1250 | Fragment Analysis |  | 6 | 32 | 7 | 99.57806 | 0.01723838 | 0.8381838 |
| GVAR1251 | Sequencing |  | 16 | 89 | 20 | 97.8903 | 0.3132847 | 0.1200429 |
| GVAR1252 | Sequencing |  | 7 | 36 | 11 | 99.15612 | 0.1407958 | 0.1007602 |
| GVAR1253 | Sequencing |  | 8 | 277 | 6 | 97.8903 | 0.05363151 | 0.3862418 |
| GVAR1254 | Fragment Analysis |  | 3 | 10 | 5 | 100 | 0.3956145 | 0.0596953 |
| GVAR1255 | Sequencing |  | 2 | 33 | 2 | 99.15612 | 0.7344313 | 0.4883052 |
| GVAR1256 | Sequencing |  | 2 | 48 | 1 | 99.15612 | 0.9784075 | 0.1728811 |
| GVAR1258 | Fragment Analysis |  | 5 | 21 | 7 | 91.13924 | 2.69E-15 | 0.4669302 |
| GVAR1259 | Sequencing |  | 10 | 400 | 11 | 95.35865 | 3.42E-05 | 0.1225503 |
| GVAR1261 | Sequencing |  | 3 | 55 | 1 | 97.04641 | 0.4796751 | 0.0959912 |
| GVAR1262 | Sequencing |  | 3 | 12 | 5 | 99.15612 | 0.579357 | 0.193552 |
| GVAR1263 | Fragment Analysis |  | 6 | 22 | 9 | 99.57806 | 7.06E-06 | 0.2111296 |
| GVAR1264 | Sequencing |  | 2 | 18 | 1 | 99.15612 | 0.7422638 | 0.0084927 |
| GVAR1265 | Sequencing |  | 2 | 20 | 2 | 97.8903 | 0.9994637 | 0.0109709 |
| GVAR1266 | Fragment Analysis |  | 4 | 13 | 7 | 100 | 3.05E-05 | 0.8601974 |
| GVAR1267 | Fragment Analysis |  | 12 | 73 | 14 | 98.31224 | 0.000107948 | 0.3456637 |
| GVAR1268 | Fragment Analysis |  | 6 | 23 | 9 | 97.8903 | 0.1849211 | 0.0497154 |
| GVAR1269 | Fragment Analysis |  | 6 | 30 | 10 | 99.57806 | 2.09E-11 | 0.0142556 |
| GVAR1270 | Sequencing |  | 2 | 23 | 1 | 98.73418 | 0.5598784 | 0.7394758 |
| GVAR1271 | Fragment Analysis |  | 6 | 27 | 11 | 99.57806 | 0.04392781 | 0.0460829 |
| GVAR1272 | Sequencing |  | 4 | 17 | 3 | 95.7806 | 0.9186529 | 1 |
| GVAR1273 | Sequencing |  | 16 | 60 | 19 | 97.8903 | 0.04507253 | 0.206269 |
| GVAR1274 | Sequencing |  | 8 | 49 | 12 | 94.09283 | 0.2384607 | 0.0936434 |
| GVAR1276 | Fragment Analysis |  | 6 | 25 | 11 | 96.20253 | 8.33E-11 | 0.2358201 |
| GVAR1278 | Sequencing |  | 3 | 113 | 5 | 99.57806 | 0.2586871 | 0.1253368 |
| GVAR1279 | Fragment Analysis |  | 19 | 152 | 27 | 97.04641 | 0.005175695 | 0.0020348 |
| GVAR1280 | Sequencing |  | 2 | 4 | 1 | 98.31224 | 0.6084612 | 0.8700865 |
| GVAR1281 | Sequencing |  | 2 | 30 | 1 | 96.62447 | 0.7419066 | 0.740985 |
| GVAR1282 | Fragment Analysis |  | 6 | 25 | 6 | 97.46835 | 2.30E-05 | 0.8646391 |
| GVAR1283 | Fragment Analysis |  | 7 | 36 | 10 | 99.57806 | 0.000234909 | 0.386983 |
| GVAR1284 | Sequencing |  | 9 | 32 | 15 | 96.20253 | 0.2324289 | 0.0681186 |
| GVAR1285 | Sequencing |  | 9 | 27 | 16 | 98.73418 | 0.6127782 | 0.3299422 |
| GVAR1286 | Sequencing |  | 2 | 103 | 1 | 98.73418 | 0.5334821 | 0.0698469 |
| GVAR1287 | Fragment Analysis |  | 3 | 10 | 4 | 100 | 0.8068797 | 0.0013927 |
| GVAR1289 | Sequencing | CGEN-40002 | 2 | 19 | 1 | 99.15612 | 0.2984737 | 7.21E-05 |
| GVAR1290 | Sequencing |  | 2 | 27 | 2 | 98.31224 | 0.5568455 | 0.706796 |
| GVAR1292 | Fragment Analysis | CGEN-40003 | 7 | 42 | 8 | 100 | 0.2587942 | 5.07E-05 |
| GVAR1293 | Sequencing |  | 2 | 26 | 1 | 100 | 0.9817292 | 1 |
| GVAR1294 | Sequencing |  | 2 | 4 | 1 | 99.15612 | 0.4942713 | 0.4699277 |
| GVAR1295 | Sequencing |  | 4 | 117 | 4 | 98.31224 | 0.02893134 | 0.2797793 |
| GVAR1296 | Sequencing |  | 3 | 58 | 3 | 100 | 0.8383423 | 0.2429 |
| GVAR1298 | Sequencing |  | 9 | 36 | 7 | 95.7806 | 0.1566386 | 0.0906897 |
| GVAR1300 | Sequencing |  | 8 | 32 | 6 | 99.15612 | 0.2138956 | 0.0420302 |
| GVAR1302 | Fragment Analysis |  | 7 | 29 | 16 | 99.57806 | 0.000417019 | 0.2545321 |
| GVAR1304 | Sequencing |  | 6 | 30 | 4 | 99.15612 | 0.4092962 | 0.1937478 |
| GVAR1306 | Sequencing |  | 4 | 9 | 6 | 96.62447 | 0.396078 | 0.4616216 |
| GVAR1308 | Fragment Analysis |  | 5 | 28 | 7 | 99.15612 | 0.1826903 | 0.211802 |
| GVAR1310 | Sequencing |  | 2 | 21 | 0 | 100 | 1 | 1 |
| GVAR1311 | Sequencing |  | 4 | 9 | 5 | 99.15612 | 0.6209919 | 0.5849486 |
| GVAR1312 | Sequencing |  | 2 | 23 | 0 | 99.57806 | 1 |  |
| GVAR1313 | Sequencing |  | 8 | 33 | 11 | 98.73418 | 0.1483358 | 0.2519849 |
| GVAR1314 | Sequencing |  | 2 | 18 | 1 | 100 | 0.6925522 | 0.4288864 |
| GVAR1315 | Sequencing |  | 2 | 9 | 0 | 98.73418 | 1 |  |
| GVAR1316 | Sequencing |  | 5 | 19 | 5 | 100 | 0.3132537 | 0.0096381 |
| GVAR1318 | Sequencing |  | 5 | 20 | 5 | 98.31224 | 0.1955364 | 0.6057462 |
| GVAR1319 | Sequencing |  | 2 | 5 | 2 | 98.73418 | 0.9603275 | 0.0106976 |
| GVAR1320 | Sequencing |  | 4 | 112 | 3 | 96.62447 | 0.7044976 | 0.702002 |
| GVAR1321 | Fragment Analysis |  | 4 | 22 | 5 | 100 | 0.5157153 | 0.1509199 |
| GVAR1322 | Fragment Analysis |  | 6 | 23 | 4 | 99.57806 | 0.7053009 | 0.7117515 |
| GVAR1323 | Fragment Analysis |  | 3 | 11 | 6 | 96.62447 | 5.01E-15 | 0.5988537 |
| GVAR1324 | Fragment Analysis |  | 5 | 25 | 8 | 96.20253 | 0.19858 | 0.0326354 |
| GVAR1325 | Fragment Analysis |  | 5 | 27 | 7 | 100 | 0.000593369 | 0.0871154 |
| GVAR1326 | Fragment Analysis |  | 7 | 32 | 12 | 99.57806 | 2.37E-07 | 0.3090003 |

Protocol: sequencing or fragment analysis; #alleles: the number of alleles for each INDEL; Ntest: number of tests that were performed for each INDEL when comparing good responders and non-responders; Length range: length difference between longest and shortest allele; Rate (%): the fraction of samples that were genotyped successfully; Hw (Hardy-Weinberg equilibrium): p-value of a chi-square test comparing the homozygotes and heterozygotes distribution. The value in the table is the minimal value for all subdivisions of the alleles into two groups; Min p-value: p-value of a Fisher's exact test comparing good responders to non-responders. The value in the table is the minimal value of all tests done for each INDEL.
